# Knowledge self-monitoring, efficiency, and determinants of self-confidence statement in multiple choice questions in medical students

**DOI:** 10.1186/s12909-020-02352-6

**Published:** 2020-11-19

**Authors:** Nahid Tabibzadeh, Jimmy Mullaert, Lara Zafrani, Pauline Balagny, Justine Frija-Masson, Stéphanie Marin, Agnès Lefort, Emmanuelle Vidal-Petiot, Martin Flamant

**Affiliations:** 1 Physiologie-Explorations Fonctionnelles, FHU APOLLO, Assistance Publique Hôpitaux de Paris, Hôpital Bichat-Claude Bernard, Université de Paris, CRI, INSERM, F-75018 Paris, France; 2 Biostatistics, Epidemiology and Clinical Research Department, APHP Hôpital Bichat, Université de Paris, IAME, INSERM, F-75018 Paris, France; 3 Intensive Care Unit, APHP, Hôpital Saint-Louis, Université de Paris, UMR 976, INSERM, F-75010 Paris, France; 4 Physiologie-Explorations Fonctionnelles, FHU APOLLO, Assistance Publique Hôpitaux de Paris, Hôpital Bichat-Claude Bernard, Université de Paris, Neurodiderot, INSERM, F-75018 Paris, France; 5 Department of Educational Sciences in the Dean’s office, Faculté de Médecine, Paris Diderot University, Paris, France; 6 Department of Internal Medicine, Assistance Publique Hôpitaux de Paris, Hôpital Beaujon, Université de Paris, iLumens Paris-Diderot Simulation Department, IAME, UMR 1137, INSERM, Paris, F-75018 France

**Keywords:** Evaluation, Students confidence, Gender confidence gap, Feedback, Efficiency

## Abstract

**Background:**

Multiple-choice question (MCQ) tests are commonly used to evaluate medical students, but they do not assess self-confidence nor penalize lucky guess or harmful behaviors. Based on a scoring method according to the appropriateness of confidence in answers, the study aimed at assessing knowledge self-monitoring and efficiency, and the determinants of self-confidence.

**Methods:**

A cross-sectional study of 842 s- and third-year medical students who were asked to state their level of confidence (A: very confident, B: moderately confident and C: not confident) during 12 tests (106,806 events). A bonus was applied if the level of confidence matched with the correctness of the answer, and a penalty was applied in the case of inappropriate confidence.

**Results:**

Level A was selected more appropriately by the top 20% students whereas level C was selected more appropriately by the lower 20% students. Efficiency of higher-performing students was higher when correct (among correct answers, rate of A statement), but worse when incorrect compared to the bottom 20% students (among incorrect answers, rate of C statement). B and C statements were independently associated with female and male gender, respectively (OR for male vs female = 0.89 [0.82–0.96], *p* = 0.004, for level B and 1.15 [1.01–1.32], *p* = 0.047, for level C).

**Conclusion:**

While both addressing the gender confidence gap, knowledge self-monitoring might improve awareness of students’ knowledge whereas efficiency might evaluate appropriate behavior in clinical practice. These results suggest differential feedback during training in higher versus lower-performing students, and potentially harmful behavior in decision-making during clinical practice in higher-performing students.

**Supplementary Information:**

The online version contains supplementary material available at 10.1186/s12909-020-02352-6.

## Background

In *Apology*, Plato relates that Socrates, seeking the meaning of wisdom, finds out that he is only wiser than others for he knows that he does not know [1]. This “Socrates Paradox” should be in everyone’s mind in particular in senior positions, in which decisions might have a major impact, such as in medicine [2, 3]. Indeed, being alert on one’s own limits is of central importance in order to avoid medical errors [4]. In the clinical setting, practitioners aware of the limits of their competence will either seek information from textbooks or websites, or ask peers and supervisors for help. While this behavior will be less harmful in patient care than making a wrong decision in a field one does not master [5], it will at the same time be less efficient than the one consisting in being confident in one’s knowledge and making the right decision [6].

Though critical in clinical practice, evaluation of confidence in knowledge is not widely used during medical training. Yet, self-assessment is not only important in clinical practice [7], but also during the process of learning [8]. Consistently, students with high self-monitoring competence might disclose better achievements during training [9].

In the past decades, several new evaluation methods have been assessed, including script concordance tests [10]. Yet, students’ evaluation still relies mostly on tests based on multiple-choice questions (MCQ) [11]. This evaluation method is widely used throughout the world because of its convenience in scoring, and its non-inferiority in literature compared to open-ended questions [12, 13]. However, pitfalls in students’ evaluation have also been reported [14]. First, some authors have hypothesized that MCQs test only one type of knowledge based on well-defined and lower-order skills [15–17] and that it promotes neither critical thinking, nor the sense of creativity and synthesis [18]. Second, compared to open-ended questions, MCQs allow a proportion of lucky guess that might improve the final grade [19, 20]. Third, answers might be a function of questions’ prevalence in questions banks, because of frequent redundancy throughout the years [21]. And last but not least, they do not provide tools that might improve students’ feedback on their knowledge and competence except once they are provided with the correct answers [22–24]. Thus, improvement is needed in using this method.

Measuring the students’ certainty in their answers in MCQs, as well as its correlation with patient safety if applied in clinical care, has been reported in a few previous publications [25, 26]. Authors reported that correct answers correlated with self-confidence, but that confident incorrect answers were more likely to lead to harmful decisions if applied to clinical practice. Few studies also took this method into account for the scoring evaluation [27, 28], based on the model developed by Gardner-Medwin and colleagues [29, 30]. Confidence-based marking might encourage careful thinking about each question the students are facing during a test and discourage lucky guess [31, 32]. After the test, critical analysis of their results should also provide them a feedback on the confidence they have in their knowledge. However, the exact determinants of confidence and its appropriateness have not yet been evaluated.

In this view, interaction between confidence and correctness can be measured directly, but can also be differentiated into two distinct parameters. Indeed, as Tweed suggested it, correctness while being confident (namely “knowledge self-monitoring”) and confidence while being correct (namely “efficiency”) are two different abilities that should be analyzed separately [33]. They might indeed reflect different information with regards to the student’s, hence future practitioner’s, behavior.

The aims of our study were first to evaluate a scoring approach of tests adjusted based on a bonus/penalty system according to the appropriateness of confidence level statement, second to describe knowledge self-monitoring and efficiency among students according to their general performance, and third to analyze the determinants of confidence and appropriate confidence in a large cross-sectional study conducted in 842 2nd and 3rd-year medical students and 12 different tests.

## Methods

The medicine degree course in France is a six-year course divided into three periods. The first period is a common health science year leading to a competitive examination in which the testing method depends on the University, usually consisting of a majority of multiple-select MCQ tests. This is followed by 2 years of basic biomedical knowledge learning, and then 3 years of clinical rotations associated with medicine learning ending up with another competitive examination consisting of a majority of multiple-select MCQ tests. The students choose their residency course according to their ranking after this test.

### Study design, setting and participants

A total of 842 s- and third-year students from 2014 and 2015 graduating class at Paris Diderot University School of Medicine, France, were included in this cross-sectional study. Twelve tests were included from different courses (general course and/or continuous assessment for: oncology, renal physiology, nephrology, endocrinology, gynecology, neurology, cardiology, quantitative biomedicine). Each test comprised 15 to 30 questions, a majority of multiple-select MCQs (M-MCQs) and some single-select multiple-choice questions (S-MCQs), all with 5 answer options. The M-MCQs consist of questions that might each have one to 5 correct answers among the 5 answer options (and consequently 0 to 4 distractors), and students are unaware of the number of distractors. S-MCQs display only one correct answer among the 5 answer options (and consequently always 4 distractors), and students are aware of the fact that only one answer option is correct. This study was therefore based on the analysis of a total of 106,806 questions. Students were ranked according to their overall grade at each test in order to determine the top 20% and the bottom 20% students.

### Assessment of confidence

Along with answers to M-MCQs and S-MCQs, students were asked to indicate their level of confidence in the correctness of their response to each question as: A) very confident, B) moderately confident and C) not confident in a separate grid. Noteworthy, confidence statement was not compulsory in this experimental phase of the new evaluation system.

### Adjustment of scores according to the appropriateness of the confidence level

The usual scoring system of M-MCQs in our medical school is as follows: one point if answers to all 5 answer options to the question are correct, 0.5 point in case of one mistake, 0.2 point in case of 2 mistakes, 0 point in case of 3 or more mistakes. Mistakes are defined as not marking a true answer option as correct, as marking a false answer option as correct, or as omitting items or answer options. For S-MCQs, either the correct answer option is selected and the score to the question is 1, or the score is 0. Test score is then calculated depending on the total number of questions to generate a grade between 0 and 20 out of 20. The score was modified on the basis of a bonus/penalty system allowing us to adjust the score for each question according to whether the declared confidence was appropriate. High confidence in correct answers was rewarded with bonus points (+ 0.2 point), whereas high confidence in incorrect answers was penalized (− 0.3 point), as described in Table 1. This system is thus beneficial to those with an accurate self-assessment, and tones down the weight of a correct answer obtained by chance. Of note, it is also beneficial for those who are not confident in an incorrect answer, on the basis of a potentially less harmful behavior in clinical practice when one is aware of the limits of his knowledge. Noteworthy, the adjusted overall test grade could potentially be outside the 0 and 20 theoretical boundaries.

**Table 1:** Bonus/Penalty system for the adjustment of the score according to appropriateness of confidence

		Level of confidence		
		A	B	C
**Unadjusted score at the question**		**Bonus/Penalty**		
**M-MCQ**	1 (all items correct)	0.2	0	−0.2
	0.5 (1 mistake)	−0.1	0.1	0
	0.2 (2 mistakes)	−0.2	0	0.1
	0 (> 2 mistakes)	−0.3	−0.1	0.2
		**Final score at the question**		
	1 (all items correct)	1.2	1	0.8
	0.5 (1 mistake)	0.4	0.6	0.5
	0.2 (2 mistakes)	0	0.2	0.3
	0 (> 2 mistakes)	−0.3	−0.1	0.2
**Unadjusted score at the question**		**Bonus/Penalty**		
**S-MCQ**	1 (correct choice)	0.2	0	−0.2
	0 (incorrect choice)	−0.3	0	0.2
		**Final score at the question**		
	1 (correct choice)	1.2	1	0.8
	0 (incorrect choice)	−0.3	0	0.2

Abbreviations: *M-MCQ* multiple-select multiple choice question, *S-MCQ* single-select multiple choice question

**Fig. 1:**
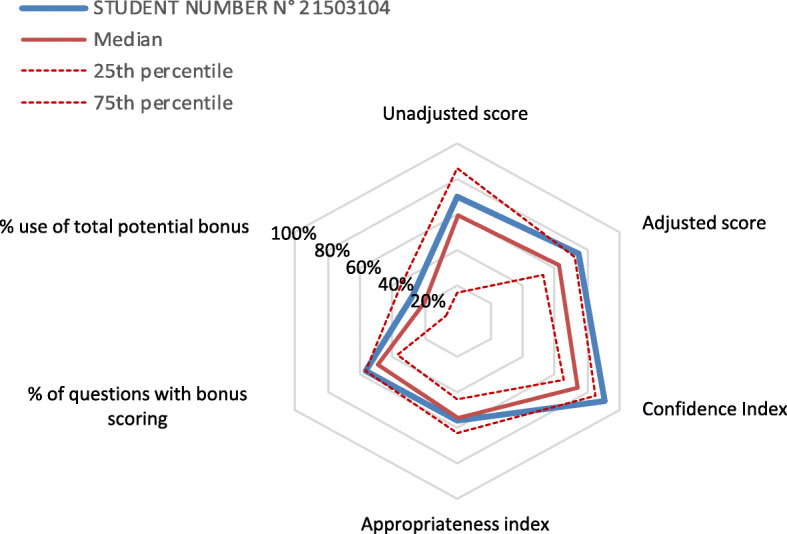
Example of an individual evaluation diagram (translated into English). Each student was given a personalized spider chart along with final results at the test. The axes are represented as relative values in percent of the maximal value. See Methods Section for the calculation of each index

This bonus/penalty scoring system was given to the students, who were therefore informed on how confidence grading would influence their scores. However, in order to obtain better adherence of students to this new testing system, the highest score (unadjusted or adjusted) was selected for annual evaluation. Along with the final results and corrections of each test, students received an individual diagram of their unadjusted and adjusted scores compared with the average score at the test, with the percentage use of total potential bonus and the highest score they could have obtained if confidence statement was always appropriate (Fig. 1). Appropriateness and confidence indexes were established, as well as overestimation and underestimation indexes, as follows:

**Confidence index** (in **%):** [n(A) + (n(B) X 0.5)] / n (questions).

**Appropriateness index** (in **%):**

M-MCQs: [n(“A/1”) + n(“B/0.5”) + n(“B/0.2”) + n(“C/0”)] / n (questions).

S-MCQs: [n(“A/1”) + n(“C/0”)] / n (questions).

(“A/1” meaning confidence level A and unadjusted score 1 etc. …)

A coefficient was applied to the M-MCQs appropriateness index and to the S-MCQs according to the rate of M-MCQs and S-MCQs in the test, and the index was then averaged.

**Overestimation index (in %):** n (points) / (n (questions) X 3).

M-MCQs: “A/0” = 3 points; “A/0.2” = 2 points; “A/0.5” = 1 point; “B/0” = 2 points; “B/0.2” = 1 point. Other = 0 point.

S-SCQs: “A/0” = 3 points; “B/0” = 2 points. Other = 0 point.

**Underestimation index (in %):** n (points) / (n (questions) X 3).

M-MCQs: “C/1” = 3 points; “C/0.5” = 2 points; “B/1” = 2 points. Other = 0 point.

S-MCQs: “C/1” = 3 points; “B/1” = 2 points. Other = 0 point.

### Satisfaction questionnaire

An anonymous questionnaire was provided to a random sample of 85 students after the exams and before the results to the exams, in order to evaluate the procedure. The questionnaire consisted of an M-MCQ survey about their general feelings regarding the evaluating system (Figure S2).

### Data analysis

Pseudonymized data regarding students demographic characteristics were collected, including gender, occurrence of 1st year repeat, type of high school degree and type of distinction. Year of university admission was recorded as a surrogate for age (birth date was a missing data). Data regarding the tests characteristics were also recorded, including the number of questions, the type of question (S-MCQs or M-MCQs), the number of correct answer option within a question in the case of MCQs, the discrimination index of the question, the average and individual grade at the test. Discrimination index of a question was defined as the mean difference of scores between students in the first tertile and students in the last tertile of the test grades.

Categorical variables were expressed as number and percentage (%) and compared using the chi-square test. Continuous variables were expressed as median and interquartile range (IQR). Multivariable analyses of determinants of each confidence degree (A, B or C, each versus the other two) and of bonus scoring were performed using a mixed logistic regression and mixed linear model, respectively, where the student-related and test-related random effects accounted for the lack of independence in the answers from the same student or answers belonging to the same test. For mixed linear regression, mean difference estimates and 95% confidence intervals (CI) were reported from asymptotic REML estimation. For mixed logistic regression, we reported odds-ratios (ORs) along with their 95% CI from asymptotic REML estimation. *P*-values for each variable in the univariate analysis were derived by the asymptotic Wald test. A *p*-value < 0.05 was considered significant. All variables with a significant *p*-value were included in the multivariable analyses, with similar test procedures. The R software version 4.6.1 with lme4 package for mixed models (lmer and glmer functions) was used for the statistical analyses.

## Results

### Descriptive analyses

Student’s characteristics were available for 734 students (366 s-year students and 382 third-year students), and are reported in Table 2. 65% were female, 70% had passed first-year exams on second attempt, 98% had a scientific high school degree, and 46% graduated with high honors. Tests characteristics are enlisted in supplementary Table S1. Twelve tests were included in the study, 4 from the second year and 8 from the third year. Median overall grades were 11.9/20 (IQR 10.1–13.1) in second-year students, and 12.5/20 (IQR 11.2–13.8) in third-year students. After score adjustment according to the level of confidence and appropriateness of confidence (see supplementary Table S1), median adjusted overall grades were higher in all tests, with a median bonus of 1.0 point (0.5–1.6).

**Table 2:** Students’ characteristics (*n* = 734 students with available demographic data)

Variable		N (%)
**Gender**	M	259 (35%)
	F	475 (65%)
**University admission year**	2016	222 (30%)
	2015	342 (47%)
	2014	124 (17%)
	< 2014	46 (6%)
**Number of first-year repeats**	0	513 (30%)
	1	219 (70%)
	2	2 (0.3%)
**High-school degree**	Scientific	721 (98%)
	Economic and Social	5 (1%)
	Other	8 (1%)
**High-school diploma distinction (*n*** **= 728)**	High honors	338 (46%)
	Second Class Honors	232 (32%)
	Third Class Honors	120 (16%)
	Average	38 (5%)

Abbreviations: *M* male, *F* female. Percentages were rounded explaining that some do not sum up to 100%

Of note, anonymous questionnaires were delivered to the students in order to evaluate the procedure. Analysis of these evaluations showed overall enthusiastic adhesion to the procedure (Figure S2).

**Fig. 2:**
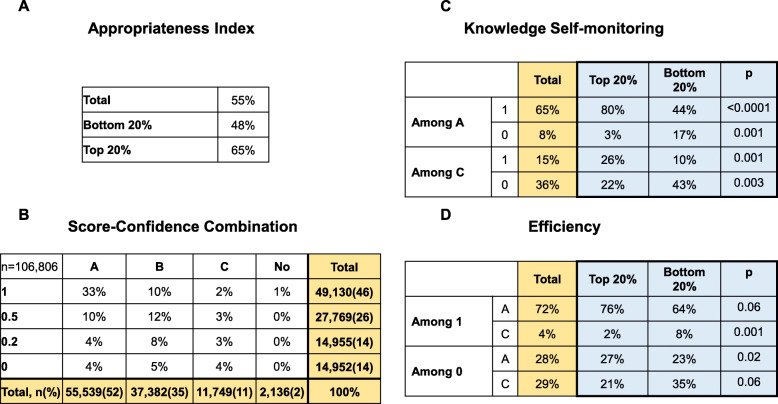
Appropriateness index, Interaction between confidence and score, knowledge self-monitoring and efficiency assessment. **a**, percentage of each combination between confidence and unadjusted score at a question in the overall population. **b**, knowledge self-monitoring, described as the percentages of scores at a question according to the level of confidence stated. The *p*-value indicates the result of the chi-squared test comparing the top 20% and the bottom 20% students. **c**, efficiency, described as the percentages of confidence statements at a question according to the level of confidence stated. The p-value indicates the result of the chi-squared test comparing the top 20% and the bottom 20% students

Data obtained for the personalized evaluation diagram (Fig. 1) allowed descriptive analyses related to confidence statement (Fig. 2a). The appropriateness score was of 55.1% in the overall population, 65.1% in the top 20% students and 48% in the bottom 20% students (*p* = 0.01 versus the top 20%). The highest level of confidence (A) was selected for 52% of the questions (Fig. 2a). Of note, the highest bonus (0.2 point, corresponding to the A-1 and the C-0 situations) was encountered more often than other situations (in 37% of the questions), with a majority of appropriate confidence in correct answers (A-1 situation, 33% of the overall questions), whereas the highest penalty (“A/0” situation) occurred in 4% of the questions. On the whole, students overestimated their knowledge in 30.5% of the cases (A statement and score < 1 point, or B statement and score <  0.5 point), and underestimated it in 12.8% of cases (B-1 situation or C statement and score > 0.2 point).

### Knowledge self-monitoring and efficiency patterns

Knowledge self-monitoring (i.e. being correct when confident) and efficiency (i.e. being confident when correct) patterns were assessed in the whole student body and compared between the top 20% students and the bottom 20% students (Fig. 2b and c). Regarding knowledge self-monitoring (Fig. 2b), among the highest confidence statement (A statement), answers were correct in 80% in the top 20% students and 44% of cases in the bottom 20% students (*p* <  0.0001), and incorrect in 3% in the top 20% students and 17% in the bottom 20% students (*p* = 0.001). Interestingly, among the C statements, answers were incorrect in 22% in top 20% students and 43% of cases in the bottom 20% students (*p* = 0.001) and correct in 26% in the 20% top and 10% in the 20% bottom students (*p* = 0.003). Consequently, the top students performed better in knowledge self-monitoring while they performed worse in self-monitoring of their deficiencies, compared to the bottom 20%.

Regarding efficiency (Fig. 2c), among the correct answers (crude score 1.0), the confidence statement was A in 76% in the top 20 and 64% in the bottom 20% students (*p* = 0.06, not significant) and C in 2% in the top 20 and 8% in the bottom 20% students (p = 0.001). Among the incorrect answers, the confidence statement was C in 21% in the top 20 and 35% in the bottom 20% students (*p* = 0.02) and A in 36% in the top 20 and 24% in the bottom 20% students (p = 0.06, not significant). Thus, although top students were more efficient when correct, they performed worse in detecting their deficiencies when incorrect.

### Determinants of bonus scoring and of confidence statements

Multivariable analysis of the determinants of bonus scoring is shown in Table 3. After adjustment, bonus scoring was independently associated with student-related characteristics such as a more recent year of admission in University (for the less recent: effect − 0.00795 [95% CI -0.01382- -0.00206], *p* = 0.042), and first-year passed on first attempt (for first-year repeating: effect − 0.00252 [95% CI -0.00478- -0.00024], *p* = 0.03). Interestingly, the “B” confidence statement was negatively associated with bonus scoring whereas the “C” statement was positively associated with bonus scoring (effect − 0.013 95% CI [− 0.01497- -0.01099] and 0.05839 [95% CI -0.0554- -0.06143] respectively, *p* <  0.0001). Test-related factors associated with bonus scoring were: a lower rate of success to the question, a higher discrimination index of the question, an M-MCQ compared to a S-MCQ, a higher number of correct answer options in the question and a higher grade at the test (respective effects − 0.0003 [95% CI -0.00035- -0.00025], *p* <  0.0001, 0.00239 [95% CI 0.00234–0.00243], p <  0.0001, − 0.03583 [95% CI -0.03984- -0.03181] for S-MCQs, p <  0.0001, 0.01721 [95% CI 0.00992–0.02449] for 5 correct items, p <  0.0001, and 0.01127 [95% CI 0.01088–0.01166], p <  0.0001). In the multivariable analysis of the determinants of the confidence statement levels, factors associated with the highest degree of confidence (A), irrespectively of its appropriateness, were only the examination-related variables stated above (Table 4), but with a positive association with S-MCQs (OR 1.589 95%CI [1.481–1.704, p <  0.0001). However, the intermediate confidence statement (B) was associated with female gender (adjusted OR = 0.89 95%CI [0.82–0.96], *p* = 0.004, Table 4), whereas the lowest confidence statement (C) was associated with male gender (adjusted OR = 1.15 [1.00–1.32], *p* = 0.047, Table 4). A fully incorrect answer with maximal confidence statement (“A/0” situation) was significantly associated with male gender in univariate analysis (OR (male vs female) = 1.2 [1–1.3], *p* = 0.0098), whereas the B-1 situation (intermediate confidence while the answer is correct) was significantly associated with female gender (OR for male vs female = 0.89 [0.82–0.97], *p* = 0.0061). This association did not remain significant in the multivariable analyses for the A-0 situation (OR = 1 [0.9–1.1], *p* = 0.99) and only a non-significant trend was found for the B-1 situation (OR = 0.95 [0.87–1], *p* = 0.23). Interestingly, the estimation of the variance of student-dependent random effect was greater than the examination-related effect in each confidence statement (data not shown), whereas the relative effect of each variable (student-related and examination-related) was broadly similar with regards to the bonus scoring.

**Table 3:** Determinants of bonus scoring (multivariable analysis)

Variable		Effect (95%CI)	p
**University admission year**	2016	0 (ref)	**0.042**
	2015	−0.00220 (−0.00587–0.00147)	
	2014	−0.00425 (− 0.00880–0.00033)	
	< 2014	− 0.00795 (− 0.01382--0.00206)	
**N 1st year repeats**		−0.00252 (− 0.00478--0.00024)	**0.03**
**Success rate of the question (%)**		−0.00030 (− 0.00035--0.00025)	**< 0.0001**
**Discrimination index of the question (%)**		0.00239 (0.00234–0.00243	**< 0.0001**
**Type of question**	M-MCQ	0 (ref)	**< 0.0001**
	S-MCQ	−0.03583 (− 0.03984--0.03181)	
**N correct answer options in the question**	1	0 (ref)	**< 0.0001**
	2	0.00758 (0.00408–0.01108)	
	3	0.01531 (0.01189–0.01874)	
	4	0.02279 (0.01887–0.02671)	
	5	0.01721 (0.00992–0.02449)	
**Unadjusted test grade**		0.01127 (0.01088–0.01166)	**< 0.0001**
**Confidence level statement**	A	0 (ref)	**< 0.0001**
	B	−0.01300 (−0.01497--0.01099)	
	C	0.05839 (0.05540–0.06143)	

Adjusted variables: gender, high-school diploma specialty, high-school diploma distinction, study year. Multivariable analysis included all the variables with a p-value< 0.05 in univariate analysis

Abbreviations: *M-MCQ* multiple-select multiple choice question. *S-MCQ* single-select multiple choice question

**Table 4:** Determinants of each level of confidence statement (multivariable analysis)

Confidence Level		A		B		C	
Variable		OR (95%CI)	p	OR (95%CI)	p	OR (95%CI)	p
**Gender**	F	–	NS	1 (ref)	0.004	1 (ref)	
	M	–		0.89 (0.82–0.96)		1.15 (1.00–1.32)	0.047
**Success rate of the question (%)**		0.998 (0.997–0.998)	0.0001	1.004 (1.003–1.005)	0.0001	1.01 (1.01–1.01)	0.0001
**Discrimination index of the question (%)**		1.038 (1.037–1.039)	0.0001	0.98 (0.97–0.98)	0.0001	0.97 (0.97–0.97)	0.0001
**Type of question**	M-MCQ	1 (ref)	0.0001	1 (ref)	0.0001	1 (ref)	0.0001
	S-MCQ	1.589 (1.481–1.704)		0.47 (0.44–0.50)		1.99 (1.80–2.20)	
**N correct answer options in the question**	1	1 (ref)	0.0001	1 (ref)	0.21	1 (ref)	0.0001
**question**	2	1.224 (1.152–1.301)		0.96 (0.91–1.02)		0.84 (0.77–0.92)	
	3	1.246 (1.175–1.322)		0.98 (0.93–1.04)		0.76 (0.70–0.82)	
	4	1.324 (1.236–1.417)		1.04 (0.98–1.11)		0.54 (0.49–0.60)	
	5	1.486 (1.309–1.688)		0.89 (0.78–1.00)		0.41 (0.30–0.57)	
**Unadjusted test grade**		1.180 (1.170–1.190)	0.0001	0.95 (0.94–0.95)	0.0001	0.80 (0.79–0.81)	0.0001

Adjusted variables: high-school diploma specialty, high-school diploma distinction, number of first year repeats. Multivariable analysis included all the variables with a *p*-value< 0.05 in univariate analysis (the variable “gender” was not included in the model for the analysis of the determinants of the “A” level of confidence as there was no significant association in univariate analysis)

Abbreviations: *M-MCQs* multiple-select multiple choice question. *S-MCQs* single-select multiple choice question

## Discussion

Our study describes a scoring system that takes into account the students’ level of confidence and whether this confidence is appropriate. We showed that medical students during second and third years were largely confident in their answers, mainly in correct answers (appropriate confidence), in particular in higher-performing students. However, we also showed that these higher-performing students tended to be more confident when incorrect than lower-performing students, suggesting potentially harmful confidence during clinical practice. We also identified distinct confidence patterns according to gender strengthening the view of a confidence gap between genders.

This evaluating method should reinforce secure behaviors since a “bonus” was applied in the case of appropriate lack of confidence in incorrect answers and since students were provided feedback on their answers to the questions. This scoring might promote recognizing one’s own weaknesses, which is an issue in a medical culture that often eschews any perception of weakness or failure [34, 35]. Yet it might be harmful or dangerous in clinical practice not to admit that one is uninformed in a particular field [36]. Developing this ability as soon as the first years of medical school is thus of great importance as it defines true expertise [37]. The benefit on future clinical behavior, although probable, is still hypothetical, whereas Gardner-Medwin et al. showed in previous studies the positive impact during college training, improving students’ study habits and helping them distinguishing between lucky guesses and true knowledge [38].

We were pleased to obtain the students adhesion to this new evaluating system, broadly satisfied in their evaluations. First, there was a higher mean grade when adjusted according to the appropriateness of confidence statement. This observation might allow positive reinforcement in examinations. Second, students declared that it helped them identifying knowledge gaps, thus promoting the need for improvement of their learning, and providing feedback on self-awareness as well as on knowledge. This was made achievable by providing the correct answers to the questions as well as the individual evaluation diagram. These reasons led our university to now consider only the adjusted grades in students’ annual evaluation.

It should also be emphasized that this system is likely to promote not only students but also teachers’ feedback. For instance, when a majority of students stated an inappropriate high level of confidence (A statement) despite an incorrect answer, a teacher should hypothesize that either the question was phrased ambiguously, or that the message was not delivered correctly during the lessons. Statistical analyses of these examinations should therefore also provide important tools to improve the lessons and the questions in a test [28].

Our findings highlight the high confidence of medical students in their answers. Indeed, the most frequent confidence statement was “A”, consistently with previous studies showing the high confidence in answers in medical and dental students [25, 33, 39]. This high confidence was more frequently associated with a correct answer. However, in line with the latter studies, situations of inappropriate confidence were not uncommon either and the absence of confidence was much less frequent. Of note, consistency between confidence statement and answer was more frequent in third-year students compared to second-year students. Our appropriateness score was also significantly higher in the top 20% students. This parameter is thus a global marker of consistency between self-awareness and the actual score.

Along with these descriptive analyses of the interaction between confidence statement and correctness, we examined two parameters described by Tweed [33]. Indeed, the degree of confidence when the answer is correct differs from correctness of the answer when the student is confident. The former reportedly represents knowledge self-monitoring, whereas the second assesses efficiency.

The patterns of these two parameters in high performers (top 20% students) provided interesting insights. The top 20% students displayed high self-monitoring safety when confident and high efficiency when correct. However, they performed poorly in knowledge gap awareness, which was all the same a rare situation as the “C” statement occurred in only 5% of cases in these students. One could thus hypothesize that these high performers are prone to feel insecure when they do not master a concept since they are not used to facing doubt, or alternatively that they might tend to report a low level of confidence with only little doubt in the correctness of their answers.

More interestingly, among the incorrect answers, these students more frequently stated the highest degree (“A”) than the lowest degree of confidence (“C”). This demonstrates that high-performing students performed worse in detecting knowledge gaps, suggesting that, while being efficient in the majority of the situations, their behavior might be more harmful in clinical practice than low-performing students in rare situations. On the whole, we found asymmetrical results in self-monitoring safety and efficiency in high-performers suggesting that these students could benefit from improved feedback in the process of learning. These findings are in line with a previous work assessing the accuracy of self-assessment in lower-performing students [40].

Importantly, the study of these two parameters (knowledge self-monitoring and efficiency) led us to consider that they might assess two very distinct behavioral frameworks.

Knowledge self-monitoring might indeed reflect the ability to evaluate one’s knowledge and correct learning process, and might represent a useful tool in the process of learning. Efficiency, instead, might be an appropriate surrogate predictor of the behavioral pattern leading to action in clinical practice. As action depends on confidence in one’s analytic process, it should reflect the expert performance resulting from this confidence in the professional area, especially in medical decision-making. Consequently, we suggest using these two parameters in the evaluation of two distinct skills: the knowledge self-monitoring assessment for the evaluation of the process of basic learning, and the efficiency in the evaluation of occupational performance. In conclusion, our study provides interesting insights on behavioral patterns that could be useful in evaluating not only the education process, but also clinical decision-making.

We were able to study the test’s characteristics as well as student’s characteristics as covariates in order to analyze the determinants of the levels of confidence and of bonus scoring. We found that examination-related variables were associated with bonus scoring and the A, B and C confidence statements. A higher discrimination index was associated with bonus scoring and the A level of confidence, as well as a higher unadjusted score at the test. Consequently, as expected, our study suggests that the highest knowledge was associated with the highest confidence [41]. S-MCQs were more associated with A and C statements, which represent the more radical confidence statements. Indeed, there is higher chance that a student answering a S-MCQ will know for sure if his answer is correct or incorrect [42].

Interestingly, a higher number of correct answer options in an M-MCQ was associated with a higher confidence statement. While M-MCQs with 2 to 3 correct answer options are recommended in French medical education, one could hypothesize that M-MCQs with a higher number of correct answer options are easier to answer, as there are fewer distractors [43].

Regarding student-related variables, the more recent year of admission in University and first-year passed on first attempt were positively correlated with bonus scoring. University year entree should reflect the age of students in a majority of cases; this is why we used it as a surrogate for age. These results suggest that younger students are more embedded and at ease in the formal education scoring system and display somehow a better understanding of what is being asked in evaluations, without necessarily having more knowledge. This phenomenon is often called *testwiseness* in literature [44–46]. As a reminder, bonus scoring may occur if the student states that he/she is uncertain when the answer is incorrect (C statement).

Finally, multivariable analyses of the determinants of the different levels of confidence displayed interesting results, especially for gender. Female gender was significantly associated with the B statement, whereas male gender was significantly associated with the C statement. There was a negative association between the B statement and bonus scoring, and a positive correlation between the C statement and bonus scoring. Moreover, the B-statement in combination with a correct answer (B-1 situation) was associated with female gender in univariate analysis, but not in multivariable analysis even though odds ratio was very close to the values of the univariate analysis. The sample of students might be too small to disclose a correlation or the relationship might be too weak to be demonstrated.

In light of our results, we hypothesize that the B statement results of a lower confidence than the C statement. Although there was no other indication than “very confident” for the A statement, “moderately confident” for the B statement and “not confident at all” for the C statement, we believe that the C statement requires better self-assessment than the B statement.

Previous studies have shown that female medical students and practitioners are less confident in their competence [47–51]. While they perform equally or better than male mates in terms of results in tests as well as in clinical practice [52, 53], they show less confidence in clinical-based evaluations [47–54]. One could also hypothesize that as soon as their first years of medical school, female students might be discouraged from exhibiting strong confidence compared to their male mates, as suggested by previous literature on gender differences in leadership and confidence [55]. In this perspective, our evaluating method should also provide better feedback in informed students who lack confidence, especially in female students as suggested in our study.

Few studies have focused on the determination of confidence in MCQs and their correlation with harmful decisions [25, 39, 40]. In the present study, we have analyzed student-related correlates as well as test-related correlates. The formers give important information regarding the student’s background in answering with confidence. The latter give clue regarding the type of examination and how it can influence the certainty with which a student will give an answer. Of note, the student-related random-effect was more important than the effect of the tests in the level of confidence stated. The inherent characteristics of the students therefore probably give them a confident or non-confident profile rather than the test itself. We were not able to identify these characteristics with the available data. This is of importance in the teaching feedback, demonstrating that though heterogeneous, we did not detect major gaps in knowledge transfer in the different tested disciplines.

Adjustment of the scores with this grading system might also reduce the part of chance in uninformed students who lack knowledge, as it benefits those who state an appropriate lack of confidence in an incorrect answer [56]. Moreover, although widely used worldwide in every domain, MCQ-based evaluations assess one type of skill that does not summarize what is needed in the future career [57]. This point will need to be addressed in further studies.

Our study displays some limitations. First, we lacked some student-related data, in particular students’ age. As University year of admission was available, we used it as a surrogate for age, though this interpretation does not take into account students with alternative educational history. Second, the evaluating method was developed in 2nd and 3rd years of medical school, based on basic science learning. Consequently, we were not able to distinguish harmful or dangerous answers and correlate them with certainty, as in studies from Tweed et al and Rangel et al [39, 40]. Our results will however hopefully pave the way to initiating this method in subsequent years of medical school. Third, we chose the bonus and penalty on the base of an arbitrary, yet collective decision. The analysis of the determinants of the different levels of confidence remain however valid whatever the bonuses and penalties would have been chosen. Besides, our study did not assess the usefulness of this approach in students’ ranking. This important issue is crucial in a French evaluating system based on a ranking at the end of the first and the sixth year of medical school [58]. According to this ranking, students will be able to enter medical school at the end of the first year, and to choose their specialty at the end of the sixth year. Moreover, the ranking system at the end of the sixth year of medical school in France displays some flaws, including the fact that it does not satisfactorily break a tie among a majority of students [59]. It should be interesting to determine if this scoring system allows a better distribution between students.

## Conclusion and perspectives

In conclusion, our study provides data regarding the determinants of self-confidence and appropriate self-confidence in M-MCQs and S-MCQs based tests in medical school. Furthermore, it might better evaluate the real capability of the students, and not only their ability to master MCQs tests [33]. This system could be useful in evaluations of medical students during their further training through knowledge self-monitoring assessment, as well as during clinical practice, through efficiency assessment. Further investigation are needed in order to evaluate the effect on personality during medical training and correlation with actual behavior during clinical practice. This mandatory hindsight on their abilities might give them healthy habits of self-criticism and searching for the knowledge they do not master.

## Supplementary Information

**Additional file 1: Supplementary Table S1.** Tests Characteristics.

**Additional file 2: Supplementary Figure S2.** Students’ evaluation of the scoring system with confidence statement. Results from a sample of 85 students.

## Data Availability

The datasets used and analyzed during the current study are available from the corresponding author on reasonable request.

## Abbreviations

M-MCQ: Multiple Select multiple-choice questions
S-MCQ: Single-Select multiple choice questions
OR: Odds Ratio
CI: Confidence Interval
