# Supplementary material for: Knowledge self-monitoring, efficiency, and determinants of self-confidence statement in multiple choice questions in medical students
Source: BMC Med Educ. 2020 Nov 19;20:445. doi: 10.1186/s12909-020-02352-6 (PMC7678098; doi:10.1186/s12909-020-02352-6)
Supplement: Supplementary file 1 — Additional file 1: Supplementary Table S1. Tests Characteristics. [file 12909_2020_2352_MOESM1_ESM.docx]

**Supplementary Table S1. Characteristics of the tests**

| **Test** | **N students** | **Non-adjusted test score**  **(median, Q1-Q3)** | **Adjusted test score**  **(median, Q1-Q3)** | **Difference**  **(median, Q1-Q3)** |
| --- | --- | --- | --- | --- |
| **1** | 391 | 16.3 (14.9-17.5) | 18.6 (16.3-20.2) | 2.2 (1.6-2.7) |
| **2** | 379 | 14.4 (12.7-16.3) | 15.7 (13.6-18.3) | 1.3 (0.8-2.0) |
| **3** | 374 | 13.7 (11.3-15.6) | 14.8 (11.9-17.3) | 1.2 (0.6-1.8) |
| **4** | 375 | 12.5 (10.6-14.3) | 13.9 (11.9-15.7) | 1.2 (0.81-1.6) |
| **5** | 394 | 10.6 (9.1-12.4) | 11.7 (9.1-13.8) | 0.73 (0.20-1.3) |
| **6** | 387 | 9.7 (8.2-11.9) | 10.6 (8.8-12.9) | 1.1 (0.47-1.5) |
| **7** | 387 | 10.5 (8.9-12.4) | 11.3 (9.1-13.5) | 0.80 (0.33-1.2) |
| **8** | 392 | 10.6 (9.2-12.1) | 11 (9.5-12.8) | 0.50 (0.07-0.9) |
| **9** | 392 | 13.1 (11.7-14.3) | 14.1 (12.3-15.7) | 0.97 (0.48-1.4) |
| **10** | 391 | 12.7 (10.8-14.3) | 13.3 (11.1-15.6) | 0.80 (0.13-1.3) |
| **11** | 391 | 11.3 (9.4-13.4) | 11.9 (10-14.7) | 0.86 (0.14-1.6) |
| **12** | 391 | 12.4 (10.7-14.3) | 13.3 (11.1-15.7) | 0.78 (0.33-1.4) |
| **Year 2** | 1,567 | 11.7 (9.8-13.6) | 12.4 (10.1-14.8) | 0.80 (0.27-1.3) |
| **Year 3** | 2,690 | 12.8 (10.5-14.9) | 13.9 (11.1-16.5) | 1.1 (0.55-1.7) |
| **Total** | 4,257 | 12.4 (10.3-14.5) | 13.4 (10.8-16) | 1.0 (0.47-1.6) |

Tests scores based on a total of 20 points.
