# Supplementary material for: Knowledge self-monitoring, efficiency, and determinants of self-confidence statement in multiple choice questions in medical students
Source: BMC Med Educ. 2020 Nov 19;20:445. doi: 10.1186/s12909-020-02352-6 (PMC7678098; doi:10.1186/s12909-020-02352-6)
Supplement: Supplementary file 2 — Additional file 2: Supplementary Figure S2. Students’ evaluation of the scoring system with confidence statement. Results from a sample of 85 students. [file 12909_2020_2352_MOESM2_ESM.pptx]

## Slide 1
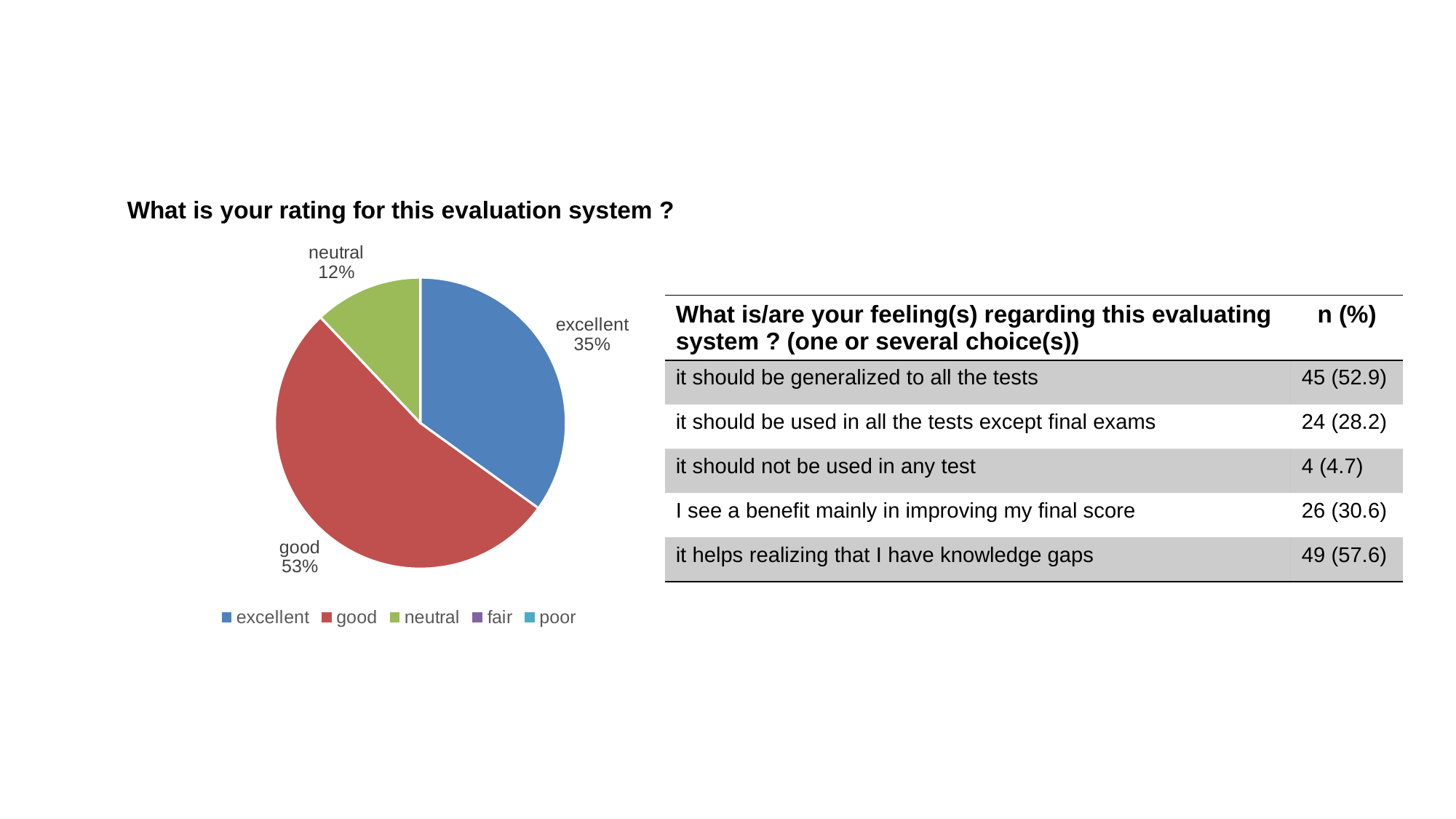

### Chart: What is your rating for this evaluation system ?
| Category | |
|---|---|
| excellent | 0.3493975903614458 |
| good | 0.5301204819277109 |
| neutral | 0.12048192771084337 |
| fair | 0.0 |
| poor | 0.0 || What is/are your feeling(s) regarding this evaluating system ? (one or several choice(s)) | n (%) |
| --- | --- |
| it should be generalized to all the tests | 45 (52.9) |
| it should be used in all the tests except final exams | 24 (28.2) |
| it should not be used in any test | 4 (4.7) |
| I see a benefit mainly in improving my final score | 26 (30.6) |
| it helps realizing that I have knowledge gaps | 49 (57.6) |
